# Wearable Based Calibration of Contactless In-home Motion Sensors for Physical Activity Monitoring in Community-Dwelling Older Adults

**DOI:** 10.3389/fdgth.2020.566595

**Published:** 2021-01-20

**Authors:** Narayan Schütz, Hugo Saner, Angela Botros, Philipp Buluschek, Prabitha Urwyler, René M. Müri, Tobias Nef

**Affiliations:** ^1^ARTORG Center for Biomedical Engineering Research, University of Bern, Bern, Switzerland; ^2^Sechenov First Moscow State Medical University, Moscow, Russia; ^3^DomoSafety S.A., Lausanne, Switzerland; ^4^Department of Neurology, University Neurorehabilitation Unit, University Hospital Bern (Inselspital), University of Bern, Bern, Switzerland

**Keywords:** sensor calibration, pervasive computing, passive infrared, physical activity, older adults, outing imputation, ambient assisted living, telemonitoring

## Abstract

Passive infrared motion sensors are commonly used in telemonitoring applications to monitor older community-dwelling adults at risk. One possible use case is quantification of in-home physical activity, a key factor and potential digital biomarker for healthy and independent aging. A major disadvantage of passive infrared sensors is their lack of performance and comparability in physical activity quantification. In this work, we calibrate passive infrared motion sensors for in-home physical activity quantification with simultaneously acquired data from wearable accelerometers and use the data to find a suitable correlation between in-home and out-of-home physical activity. We use data from 20 community-dwelling older adults that were simultaneously provided with wireless passive infrared motion sensors in their homes, and a wearable accelerometer for at least 60 days. We applied multiple calibration algorithms and evaluated results based on several statistical and clinical metrics. We found that using even relatively small amounts of wearable based ground-truth data over 7–14 days, passive infrared based wireless sensor systems can be calibrated to give largely better estimates of older adults' daily physical activity. This increase in performance translates directly to stronger correlations of measured physical activity levels with a variety of age relevant health indicators and outcomes known to be associated with physical activity.

## Introduction

Population aging poses unprecedented global challenges to modern health care systems, economies and last but not least, society as a whole (1, 2). Modern information and communication technology has the potential to contribute in overcoming some of these challenges (3–5). This includes the use of pervasive computing technology, such as microprocessor enhanced objects of everyday life. Small sensing devices like smartwatches or smart home appliances may be used to provide continuous remote monitoring of relevant health indicators and outcomes (4), increasingly referred to as digital biomarkers (6–8). These may allow for early detection of health deteriorations, enabling for instance better preventive measures or earlier interventions (9, 10). Additionally, monitoring of relevant digital biomarkers by means of pervasive computing technologies could allow for continuous assessments of chronic conditions and help in evaluating intervention efficacy (9, 11).

Physical activity (PA) is associated with a wide range of health benefits, including lower rates of all-cause mortality, non-communicable diseases, cardiorespiratory and muscular fitness across all age groups. Regular PA also helps to protect against frailty, sarcopenia, and cognitive decline (12–14). Wearable technologies, known as wearables, that can track individual's PA behavior are popular consumer items with a worldwide distribution, particularly in younger and middle-aged populations. Also, wearable accelerometers are a well-accepted method to objectively measure PA in everyday life (15–17).

While wearable devices like smartwatches, smartphones or fitness trackers would be ideal to track a variety of health relevant markers like physical activity, post-implementation based experience, including our own, point toward a clear preference for unobtrusive contactless sensing devices (9). Reasons for that may include a certain social stigma associated with visibly wearing devices amongst peers (18, 19), difficulty in handling them, added discomfort of having to think about charging and wearing a device (20), as well as skin irritations related to long-term biosensor wear (intensified by sweat in summer). While some of the mentioned issues are related to the perception of the current generation of older adults toward technology, handling wearable devices that need regular maintenance, can also be problematic for older adults with motor, cognitive, and especially memory related, issues. However, the alternative, wireless ambient sensors, are oftentimes either less accurate (for instance infrared sensors or bed motion sensors) or overly intrusive (for instance video or audio-based recording devices).

The use of wearable devices for initial calibration of less accurate but unobtrusive ambient sensors for PA quantification is a novel approach that could minimize the burden of wearing a device, while improving the reliability and thus usefulness of unobtrusive ambient sensors for physical activity tracking significantly. A similar strategy was employed with passive infrared (PIR) sensor based gait-speed estimation, where calibration was performed using a sensor array as ground-truth, but as the authors state, another source, such as a wearable device, could have been used (21).

PIR motion sensors are rather inexpensive, contactless, and unobtrusive. Therefore they are commonly used in long-term in-home monitoring settings with older adults (9, 11, 22–26). We have previously shown that in-home physical activity, quantified by PIR motion sensors can be used to approximate physical activity in old and oldest-old community-dwelling adults (26). However, the PIR motion sensor-based approach has two main disadvantages: (1) baseline activity comparisons of absolute values between participants are difficult if apartments and sensor placements differ and (2) it is unclear how to address outings correctly. We aim to address both problems by using the much more accurate and well-validated accelerometer based physical activity, to initially calibrate the ambient sensor systems.

## Methods

### Participants

The data used for this work stems from a study where modern pervasive computing systems were evaluated for telemonitoring in older adults (26). Participants were part of the StrongAge cohort in Olten (Switzerland) (27) and should represent a naturalistic population sample of community-dwelling, alone-living, old and oldest-old adults in Switzerland. We included all participants that had at least 60 days of wearable activity data recorded (first 30 days reserved for calibration and ≥30 days for evaluation) in this analysis, totalling 20 participants (age = 88 ± 8 years). The 60 days were chosen to include as many participants in the dataset as possible while guaranteeing a minimal number of data points.

The original study was conducted based on principles defined in the Declaration of Helsinki and approved by the Ethics Committee of the canton of Bern, Switzerland (KEK-ID: 2016-00406). All subjects signed and handed in an informed consent before study participation.

### Pervasive Computing Systems

In this work we made use of the DomoCare® home monitoring system for older adults (DomoSafety S.A., Lausanne, Switzerland), the same as in (26). The system consists of PIR motion sensors (sampling at 0.5 Hz) placed in the participant's apartment. Kitchen, toilet, living-room, entrance, and bedroom were always equipped with at least one sensor, if a separate bathroom was present it was equipped with a sensor as well. In addition, a magnetic door sensor was placed on the entrance and fridge door, respectively. All sensing units communicate via the ZigBee protocol with a base unit, that then sends data to a secure cloud in real-time. The PIR system allows motion detection in individual rooms based on changes in infrared radiation caused by human activity (28). The door sensors allow outings to be calculated based on entrance door opening and closing events, as explained in (29).

For the calibration of the PIR sensor system we used the medical grade Everion® biosensor worn on the upper arm (Biovotion AG, Zürich, Switzerland). Amongst other sensors, the device contains a 3-axis accelerometer that samples at 50 Hz and outputs/stores aggregated and standardized activity (vector magnitude) at 1 Hz. The participants wore the device throughout the daytime and put it on an inductive charger overnight. While charging the device, data was transmitted to a smartphone via Bluetooth Low Energy which was then encrypted and automatically transferred to a secure cloud. Data from DomoCare® systems was first stored on cloud instances from DomoSafety S.A. located in Switzerland and data from the Everion® was initially stored on instances at the University of Bern. Post collection, all data was subsequently transferred to local servers and ingested into an OmniSci (OmniSci, San Francisco, CA, United States) analytics database instance after quality control. A schematic including the data structure is available in the Appendix. To initially ensure accelerometer validity, we compared values from the Everion® with the widely used and validated (30) Axivity AX3 (Axivity Ltd., Newcastle, UK), 3-axis accelerometer [calibrated to local gravity and temperature, as described in (31)] and found good overall agreement.

### Problem Definition

There are three major limitations related to the use of PIR sensors for PA quantification: (1) Motion measured by the commonly used simple PIR motion sensors is converted to a binary response, zero if there was no change in infrared radiation above the sensor's sensitivity threshold and one otherwise. It is thus apparent, that simple PIR motion sensors cannot differentiate between the intensity of the motion, unlike a body attached accelerometer; (2) the angle and distance to a sensor can influence if and how long motion is being detected; (3) the size of equipped rooms and the apartment layout in general can lead to different results for the same amount of physical activity exerted by a person. As a result, even if the same person performed the exact same finite set of activities *A* = {*a*_1_, …, *a*_*n*_} in different PIR motion sensor equipped apartments, measures of these activities between the PIR motion sensor measurement functions *f*_*PIR*_:*A* → *M*_*PIR*_; *M*_*PIR*_ ∈ℝ_+_ and the accelerometer *f*_*acc*_:*A* → *M*_*acc*_, *M*_*acc*_ ∈ ℝ_+_ would likely differ widely. Now in reality, this simplification is not exactly true, because certain activities *a*_*i*_ will be measured by the accelerometer but not by the PIR sensors—for instance when a person is outside the apartment, outside the field of view of the PIR sensors or in a non-equipped room. This gives rise to a subset of all measured activities Ã ⊆ *A* = { *a*_*i*_ | *a*_*i*_ ∈ *A, a*_*i*_ ∈ *dom*(*f*_*PIR*_)} . We will henceforth refer to *f*_*acc*_ that is only defined over this subset as ${\tilde{f}}_{acc}:Ã\to \;{\tilde{M}}_{acc}$.

The idea of initial calibration is then to find a mapping ${\hat{f}}_{PIR}:Ã\to {\hat{M}}_{PIR}$, such that for a given activity *a*_*i*_, the Euclidean distance between the calibrated PIR motion measurement function ${\hat{f}}_{PIR}$ and the domain restricted accelerometer measurement function ${\tilde{f}}_{acc}$ is minimized, which can be thought of as a classic regression objective:


$$\begin{array}{l} \min\sqrt{{\left({\hat{f}}_{PIR}(a_{i})-{\tilde{f}}_{acc}(a_{i})\right)}^{2}}\;\forall \;i=1\ldots n \end{array}$$

Well-calibrated ${\hat{f}}_{PIR}$ functions from different PIR sensor equipped apartments should then allow that somewhat similar results are obtained for a given activity, since *f*_*acc*_, given a certain activity *a*_*i*_ should be similar across apartments. This assumption is only true if the difference between ${\tilde{f}}_{acc}$ and *f*_*acc*_ is not too large and the accelerometer intensity measurements between participants is mostly comparable. The latter assumption is likely true as for instance described in (32), while the former is largely apartment and person specific but may be improved upon by including an estimate for activity while outside.

### Learning Calibration Function

To find a suitable function ${\hat{f}}_{PIR}$, or in this case a distribution over ${\hat{f}}_{PIR}$ we propose to use Gaussian process regression, such that ${\hat{f}}_{PIR}\;\sim \;GP\left(\mu ,k\right)$, where μ(*A*) = 0 is the standardized activity mean and k(A, A′) the activity covariance function. Gaussian process regression (GPR) provides various characteristics that are likely useful in our calibration scenario. First, it allows non-linear relationships to be modeled and is non-parametric (33). In addition, GPR is known to work well with relatively little data and allows a predictive distribution to be obtained, which can help in detecting model uncertainty (33, 34). The included epistemic model uncertainty could be helpful post calibration as it could allow for quantification when patterns not seen during calibration occur, and give respective warnings if total uncertainty increases.

In the shown experiments we ended up using $k\left(a_{i},a_{j}\right)=\sigma_{0}^{2}+a_{i}\cdot a_{j}+\sigma_{n}^{2}\delta_{ij}$, as kernel defining the covariance function, where δ_*ij*_ is a Kronecker delta, $\sigma_{n}^{2}$ is a learnable bias term and $\sigma_{0}^{2}\;$ is a learnable noise constant representing additional homogenous aleatoric uncertainty in the activity measurements (33). To give a comparison how other, more traditional algorithms might perform, we additionally evaluated calibration performance with a regular linear regression (LR) algorithm and the popular XGBoost (XGB) implementation (35) of a tree boosting algorithm. The GPR kernel and hyperparameters for the other algorithms were selected by means of 3-fold cross-validation (splitting at the participant level) and random search (36). It is rather difficult to assess the usefulness of the predictive distribution, obtained by the marginal normal of the GP, in a realistic manner. We try to quantify its utility by calculating the linear correlation between the daily average $MAE_{p}^{d}$ (see below) and the daily average uncertainty estimate (the σ of the marginal Gaussian distribution).

### Data Pre-processing and Representation

We represent individual activities *a*_*i*_ as activity bouts/islands and describe their characteristics in vector space. The activity islands are extracted by first applying a simple moving average low pass filter, with 1-min length, to the total PIR motion activity signal (the sum of the duration where all PIR motion sensors in an apartment were active) and then extracting the activity islands (stretches where low-pass filtered activity is constantly > 0). Based on these islands we calculate the following features that can be used to summarize the islands in vector space: the total duration of the island, the hour of the day, the duration PIR sensors detected activity for each equipped room and the relative activity of each room with respect to the total island duration. Corresponding activity from the wearable accelerometer was also extracted and summed over the island duration, giving us the target activity *f*_*acc*_. The feature matrix was standardized to have zero mean and unit variance across column.

### Statistical Evaluation Metrics

First, it should be noted that evaluation was always performed on all available data beyond the initial 30 days that were reserved for calibration. Throughout this work we refer to multiple evaluation metrics that are explained here. First, the mean absolute error *MAE*_*p*_ between the activity estimate for each activity island and its corresponding accelerometer activity was calculated for each participant *p*. $MAE_{p}^{d}$ refers to the same but averaged over a day *d*. Second, to measure the proportionality of calibration, the Pearson correlation coefficients ρ_*p*_ between the sum of estimated post calibration activity ${\hat{F}}_{PIR}^{d}=\underset{a_{i}\in d}{\sum }{\hat{f}}_{PIR}\left(a_{i}\right)$ per day *d* and the sum of total accelerometer activity $F_{acc}^{d}=\underset{a_{i}\in d}{\sum }f_{acc}\left(a_{i}\right)$ per day *d* were calculated. Similarly to ρ_*p*_, we calculate ${\tilde{\rho }}_{p}$ the Pearson correlation coefficient between the sum of daily calibration activity ${\hat{F}}_{PIR}^{d}$ and domain restricted sum of accelerometer activity ${\tilde{F}}_{acc}^{d}=\underset{a_{i}\in d,a_{i}\in Ã}{\sum }{\tilde{f}}_{acc}\left(a_{i}\right)$. For all metrics, the sample average over all participants can be calculated, resulting in the global *MAE*, ρ and, $\tilde{\rho }$.

### Determining the Amount of Wearable Ground-Truth Data

To assess the relationship between wear-time and calibration performance, we performed calibration with 1 day, 7 days, 14 days 21 days, and 30 days of accelerometer data and calculated $\tilde{\rho }$ and *MAE* for each wear-time point and each learning algorithm (as results may be algorithm dependent).

### Evaluation of Post-calibration Performance Evolution

One of the main concerns about this kind of calibration procedure is the potential degradation that calibration quality could be subjected to over time as a result of a shift in the data generating distribution (e.g., as a result of changing behavior or seasonal patterns). To assess the potential for degradation, we calculated the weekly average *MAE* for all participants over thirty consecutive weeks (if data was available). To ensure similar scales, we first standardize weekly averages by removing the median and scaled data with respect to the interquartile range. Finally, for each week, the global average was taken, and a regression line estimated. The *p*-value of the slope coefficient was then used to determine whether the parameter differed significantly (based on α = 0.05) from 0, allowing one to decide whether any relevant trend might be present.

#### Effect of Calibration on Correlations With Clinical Assessments

To evaluate how calibration influences overall relationships with health indicators and outcomes, we calculate the non-parametric Spearman's rank correlation coefficients *r* between median daily total activity and the mean of the respective clinical assessments (if multiple were taken per participant over the same duration). Clinical assessments include: the fall-risk focused Timed Up and Go (TUG) (37), the balance and gait focused Tinetti Performance Oriented Mobility Assessments (POMA-b and POMA-g) (38), the late life depression focused Geriatric Depression Scale (GDS) (39), the cognition focused Montreal Cognitive Assessment (40), the frailty focused Edmonton Frail Scale (EFS) (41) as well as muscle force focused handgrip, hip flexor and knee extensor strength. To assess whether there are statistical differences between pre- and post-calibration, we apply the non-parametric Wilcoxon signed rank test to the absolute correlation values under the alternative hypothesis that post-calibration values are on average greater compared to pre-calibration values.

### Time Spent Outside

To assess overall PA, time spent outside the home needs to be considered. In our case, using PIR in-home sensors, PA outside the home can be seen as blocks of missing data.

A common strategy to deal with missing data is called imputation, which refers to replacing missing data with substitutes (for instance a variable's mean over all observed values) (42). Imputation can often work reasonably well, if the data is “missing completely at random” or “missing at random” (42). Given that outing likely involves more physical activity than being inside, it may be impossible to correctly impute physical activity of outing periods. Fortunately, access to calibration data from a wearable (given the wearable is also worn outside, which is true in our case), allows us to estimate a factor τ_*p*_ (for each participant *p*) by which the expected inside activity should be multiplied with. To calculate this factor, we first calculate outings according to (29) and then for each outing we divide the physical activity measured by the accelerometer with the average activity of the accelerometer during the same time of day, when the person was at home. Eventually, the median of these ratios gives us τ_*p*_. A global factor τ can then be calculated by averaging over all individual participant's τ_*p*_. As we are dealing with missing time blocks, we use temporal means—similar to what has been used for imputing non-wear time intervals with accelerometers (17, 43). That is, the expected activity sum for the given time-interval (when the outing occurred) over all observed days. To evaluate the effect of this imputation procedure on overall calibration, the evaluation metric $\tilde{\rho }$ is calculated using (1) temporal mean imputation, (2) temporal mean imputation with factor τ_*p*_ and (3) temporal mean imputation with factor τ.

All data processing, analyses and plotting have been performed with the Python (Python Software Foundation) scripting language (version 3.7). For the LR and GPR algorithm implementations from Scikit-learn library (44) were used. In case of the XGB algorithm, the official Python implementation was used.

## Results

### Calibration Results With Differing Amounts of Data and Learning Algorithms

**Figure 1:**
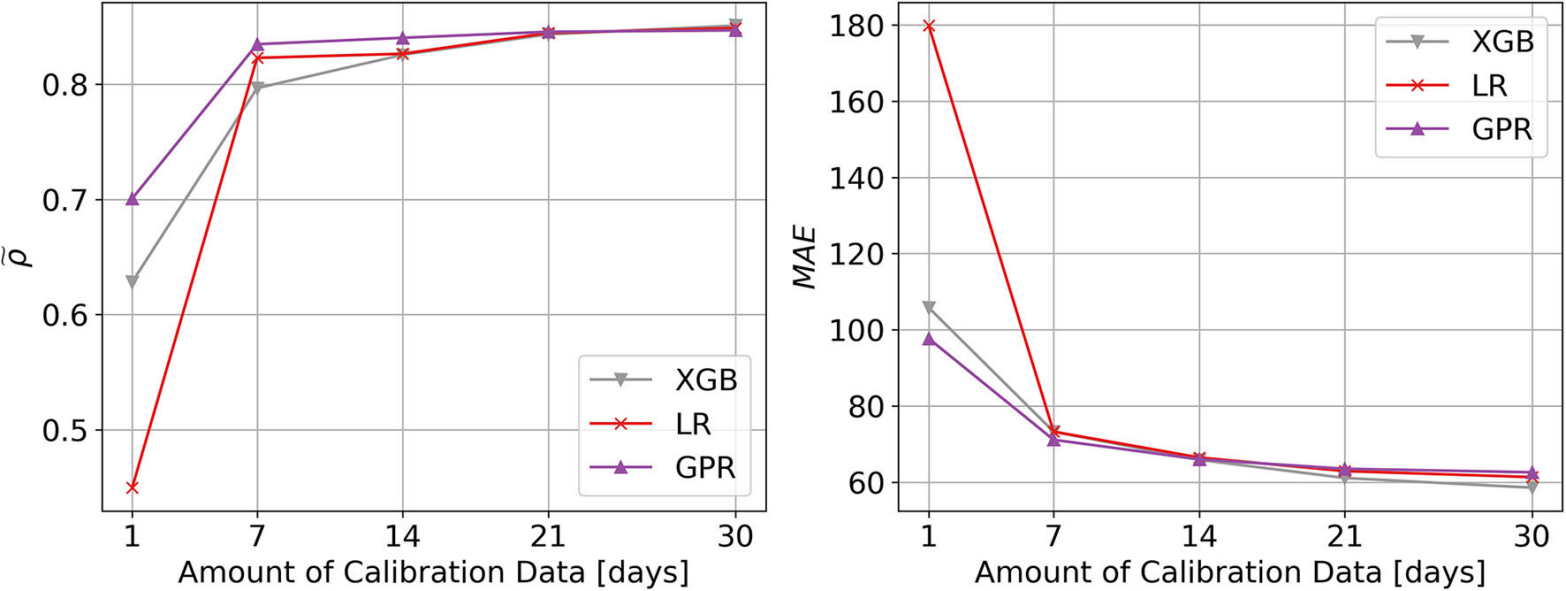
Visualization of data and algorithm dependent calibration performance. Performance of algorithms used for calibration of passive infrared sensor systems, with respect to physical activity measured by wearable accelerometers. The learning curves show the performance across all 20 participants of the calibration method against the number of days of accelerometer reference data. The different line colors show different learning algorithms used for calibration (LR, Linear Regression; XGB, XGBoost; GPR, Gaussian Process Regression). The left plot shows the Pearson correlation coefficient $\tilde{\rho }$ as evaluation criterion (higher is better), while the right plot shows the mean absolute error (*MAE)* evaluation criterion (lower is better). With GPR, only 7–14 days of reference accelerometer data is necessary to obtain a mapping quality, which can only be marginally improved upon with additional data.

In Figure 1, we visualized evaluation metric $\tilde{\rho }$ and *MAE* for 1, 7, 14, 21, and 30 days of calibration data in combination with the proposed GPR based calibration as well as LR and XGB based calibration. It should be noted, that for both evaluation metrics, the largest increase in performance can be seen between one and seven days of calibration data (from wearable accelerometer). Beyond 14 days, more data leads to increasingly smaller improvements. In case of the correlation coefficient ρ performance saturation seems to be reached by 21 days, while in case of the *MAE* saturation is not completely evident, even after 30 days. In terms of the learning algorithms used to approximate the activity calibration function ${\hat{f}}_{PIR}$, it is visible how GPR shows the best performance with little data up to 14 days. After that, GPR is mostly on par with LR and starts losing in comparison to XGB. Correlation values ρ show an average of 0.84 after 14 days. Note that all results displayed downstream were based on the 14 days calibration data case.

### Post-calibration Performance Evolution

**Figure 2:**
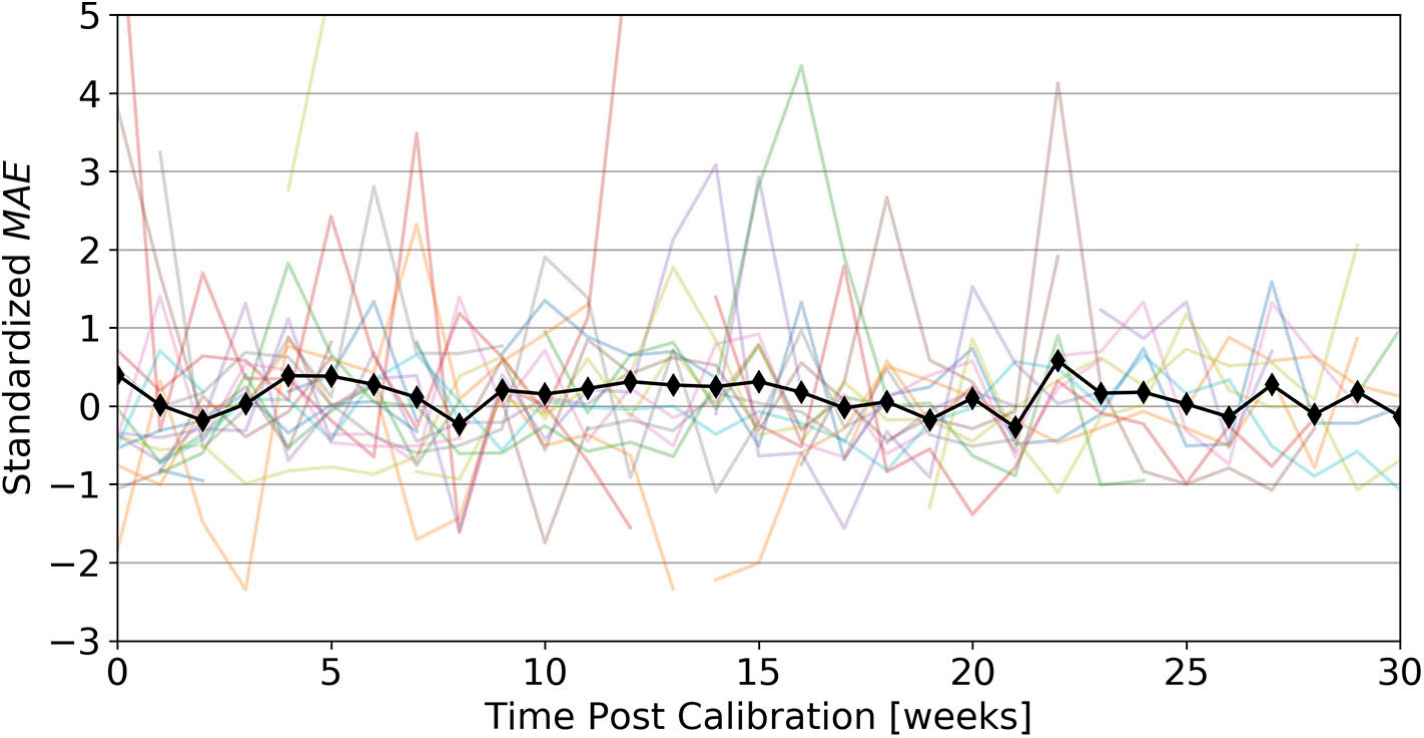
Post calibration performance evolution. shows evolution of average calibration performance, up to 30 weeks post calibration. Individual colored lines represent standardized $MAE_{p}^{d}$ for a given week of each participant, while the black line represents the standardized ***MAE*** over all participants for a given week We observe that even up to 30 weeks (~7 months) post-calibration an initial calibration using 14 days of accelerometer data remains valid.

Visually, it is difficult to discern any sort of overall deterioration throughout 30 weeks post calibration, beyond some short-term variation (see Figure 2). Regression analysis of *MAE* against time, further reveals that the slope is not statistically significant (*p* = 0.262).

### Impact of Calibration on Age Relevant Health Indicators and Outcomes

Results showing correlations of clinical assessments using calibrated and uncalibrated activity from the ambient sensor system as well as the accelerometer, demonstrate how calibration leads to increases in correlation for all assessments except hip extensor strength. Oftentimes post-calibration correlations reach strengths close to the accelerometer gold standard (see Table 1). Results based on the Wilcoxon signed-rank test, additionally suggest that the differences in correlations between pre- and post-calibration are statistically significant (*n* = 8, *p* = 0.004).

**Table 1:** Participant characteristics and demographics.

	**Pre-calibration**	**Post-calibration**	**Accelerometer**
TUG	−0.42	−0.57	−0.54
POMA–b	0.51	0.66	0.66
POMA-g	0.49	0.56	0.65
GDS	−0.43	−0.61	−0.64
MoCA	0.68	0.85	0.80
EFS	−0.56	−0.64	−0.65
Handgrip right	0.39	0.49	0.48
Hip right	0.37	0.36	0.34

*Shows Spearman's rank correlation coefficient r_s_ between clinical assessment results and median daily activity sum of the ambient system, pre- and post-calibration, as well as from the worn accelerometer sensor*.

### Handling Outings

**Figure 3:**
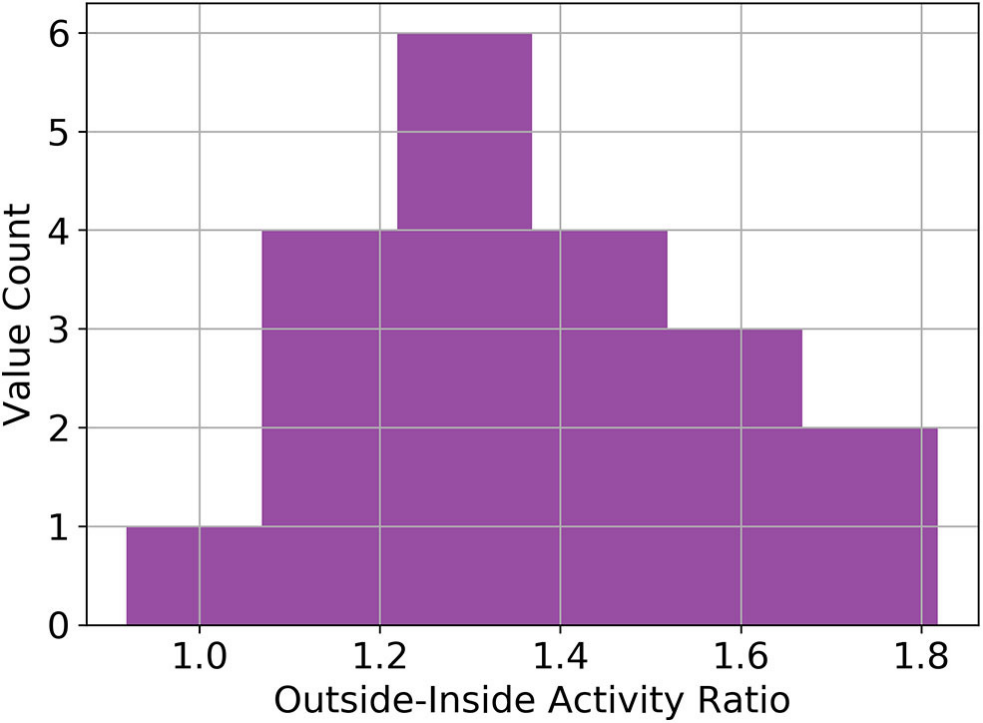
Distribution of inside-outside activity ratios. Histogram of the ratio between time spent outside and inside the home. The average value is 1.38 across all included participants. These values are based on data from a wearable accelerometer sensor.

We found that for most participants time spent outside the house leads to more activity compared to the average of the same time they spent at home. On average the ratio of activity outside vs. inside was found to be 1.38. However, depending on the person this ratio can be quite a bit different, ranging from 0.92 up to 1.82. The distribution is visualized as a histogram shown in Figure 3.

**Figure 4:**
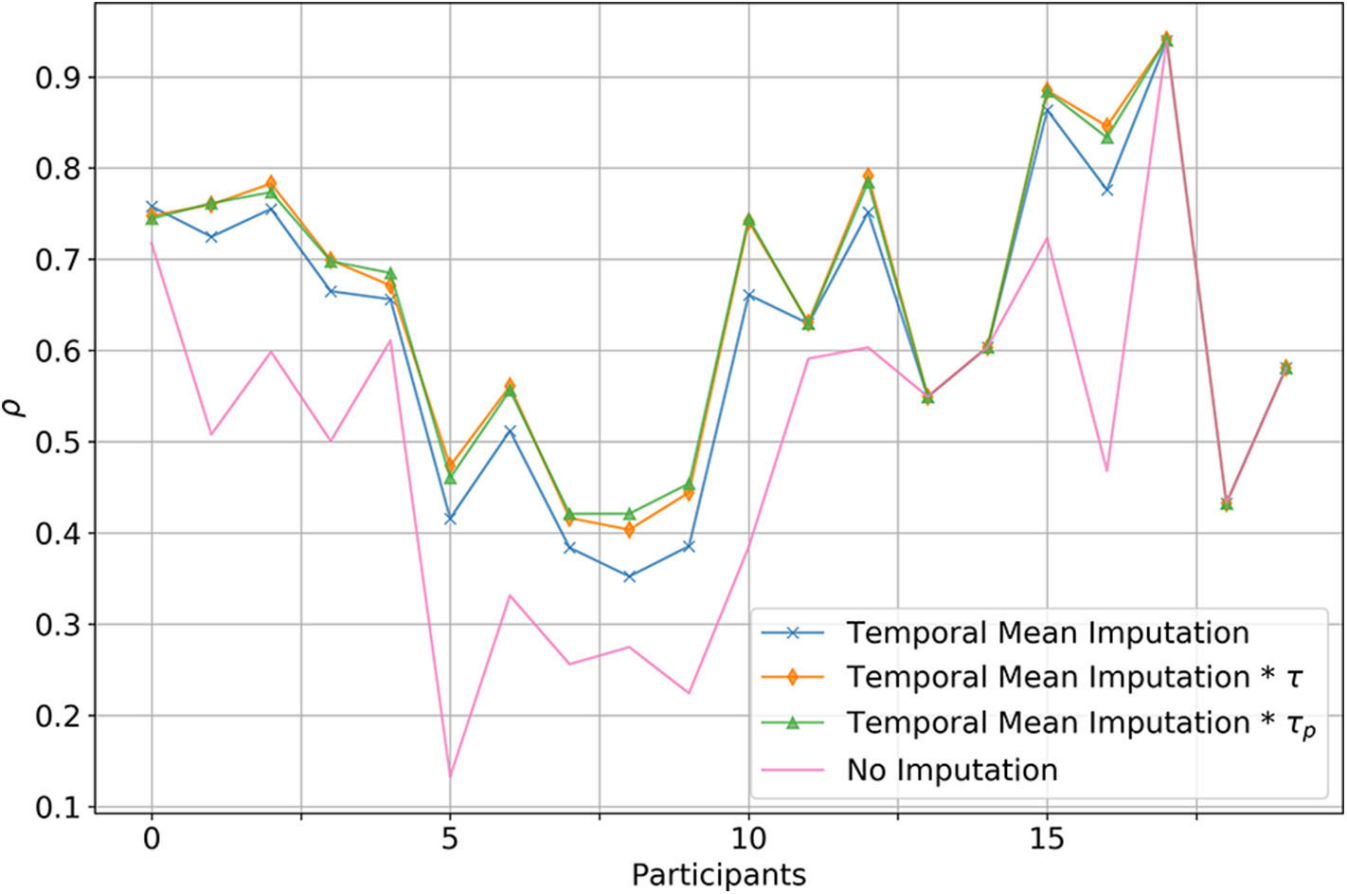
Comparison between multiple imputation strategies to handle outing. Displayed is the correlation between the total daily calibrated activity and the total accelerometer activity. In the case of the blue line, simple temporal imputation has been used to substitute missing physical activity due to outings. The orange line denotes the case where in addition to temporal mean imputation a global correction factor was added, whereas with the green line a person specific correction factor was used. Red denotes the baseline, where outings where not imputed at all.

We further found that by temporal mean imputing, ρ (the correlation to overall daily accelerometer activity) increases in most cases. Regarding the type of temporal mean imputation, using no coefficient seems to lead to significantly lower correlation values compared to using a person specific coefficient (*p* = 0.0007) or a static coefficient value (*p* = 0.0007), between which no significant difference (*p* = 0.8) was found (see Figure 4).

### Predictive Distribution

**Figure 5:**
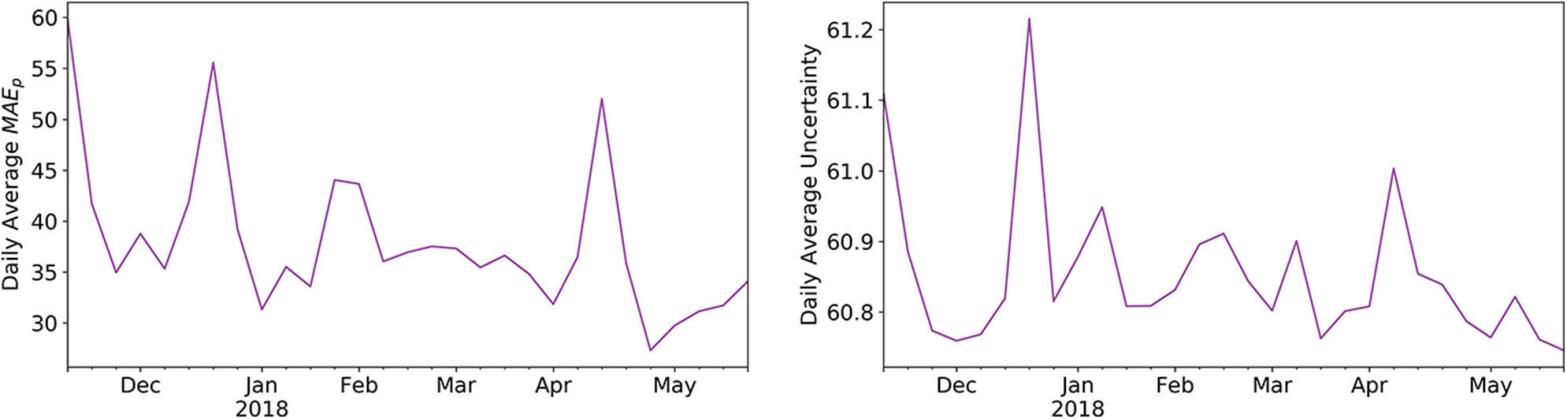
Correlation between daily average mean average errors and predictive uncertainty. Shown is the example of a participant, where we plotted the daily average ${MAE}_{p}^{d}$ and the daily average predictive distribution (both mean aggregated on a week level) as given by the marginal normal of the GPR.

To evaluate the potential usefulness of a predictive distribution we assessed how well it correlates with the daily $MAE_{p}^{d}$ for each participant. The median correlation coefficient across all participants was 0.49 ± 0.15 (min = 0.1, max = 0.67). An example of a decent correlation is given in Figure 5.

## Discussion

We found that using even relatively small amounts of wearable based ground-truth data, PIR based wireless sensor systems can be calibrated to considerably improve estimates of older adults' daily physical activity. We could additionally verify, that this increase in performance directly translates to stronger correlations of the measured physical activity levels with a variety of age relevant health indicators and outcomes, known to be associated with physical activity. This indicates that the performance gained by calibration is not only present on paper but also manifests itself in physical activity readings that capture relations to health significantly better than would be the case without calibration.

Deciding on the necessary amount of wearable data, sufficient for calibration, is a rather subjective and task specific matter, as it is a trade-off between calibration performance and wear-time. In our case, calibrating a PIR motion sensor system, 7–14 days seem to give reasonable results, with diminishing additional benefit employing longer calibration periods. We also observed that the optimal type of algorithm, approximating the calibration function ${\hat{f}}_{PIR}$, seems dependent on the amount of available calibration data. For small amounts of calibration data, GPR may be considered the best choice—which is a known property of GP based approaches (45). As a side note, in our case a linear kernel proved to be the best parametrization, which would be equivalent to using a Bayesian linear regression algorithm, but the GP view might still be more effective given little data (46). This also explains why the GPR results largely converge to the LR results given more data. On the other hand, the XGB algorithm leads to slightly better performance, given more than 14 days of calibration data, which would be the expected behavior for an algorithm with much more learning capacity. Now, since we want to restrict the necessary wear-time to a minimum, GPR is, as we initially assumed, a suitable algorithm for the task. An additional benefit of GPR's Bayesian nature, is the included predictive uncertainty, which we think can be quite useful as it often indicates a simultaneous increase in model error and may thus be used to diagnose when a calibration model's performance degrades. For our data (see Figure 5), however, we found no significant degradation in calibration performance up to 30 weeks post calibration, indicating that calibration is overall relatively stable and resilient toward smaller potential perturbations.

It comes as no surprise that it is important to somehow factor in the time spent outside, else, physical activity of people spending a lot of time outside would be vastly underestimated. The question, as how to best deal with outings in this scenario does however remain open and we did not find any work assessing this in community-dwelling old and oldest old adults. Our findings suggest that just replacing time spent outside with the average activity throughout a given time-interval is a valid strategy, leading to significant calibration improvements but does in most cases underestimate physical activity as old and oldest-old adults tend to be more physically active when outside. We found our participant population to be, on average, 1.38 times as physically active when outside, compared to if they were inside at the same time of the day (see Figure 3). Using this knowledge, it is possible to further improve outing imputation, correcting somewhat for the bias caused by outing. Interestingly, no improvements were seen between using a static global factor and employing a person specific factor, suggesting, that even if no accelerometer ground-truth was available, outings may be corrected by a factor of around 1.4. We are not exactly sure why this is, but it may be due to the fact that we are using a very rough estimate anyways and the exact factor would only have an effect if our estimates were more accurate. However, this finding merits further investigation in different populations and under varying circumstances.

Using short-term data from a more accurate wearable device seems to work well for calibrating wireless PIR ambient sensor systems. Given that previous research on the calibration of PIR sensor systems to measure gait-speed also led to very promising results (21), such relatively simple initial calibration procedures should be considered in future long-term telemonitoring applications and research employing wireless PIR sensors.

After all, our calibration procedure has its obvious limitations and problems. In general, it should be noted that due to the relatively small sample size, generalization of our results involving statistical inference may be limited. Regarding the calibration procedure, most PIR sensors have relatively low sampling rates due to the having a refractory period and a restricted field of view. This makes it virtually impossible to get a completely accurate estimate of the real physical activity, as we would get by using a high-frequency accelerometer. This means that there will likely always be a certain underestimation of physical activity even after calibration, as certain activities are just missed by the PIR system. Further, we should add that the approach can only function if someone is living alone. Although some work suggests PIR installations may be usable in a multi-person setting, this is likely not the case with physical activity quantification. Another problem is variance in results between participants (as can be easily seen in Figure 4). For certain people it did not seem possible to get a good calibration (although still slightly better than baseline), and even after in-depth manual investigation, in two instances we did not find any reasonable explanation for this behavior. Possible explanations could be that there were not enough sensors in a room, that the sensors were not placed ideally, or that the person's behavior makes it inherently difficult to capture physical activity using PIR sensors—for instance someone that is regularly taking care of the neighbor's pet. This is another important argument in favor of using reliable data for calibration of wireless systems. By employing cross-validation it is straight forward to identify installations for which there is a large disagreement before and after calibration, this also allows to manually check for potential biases using Bland-Altman plots. Considering medical applications, the validity of data coming from non-invasive ambient motion sensors is of particular importance for building up trust with this new technology, and may in that way allow for broader application. We would thus advice work related to contactless health monitoring to use more accurate and validated wearable devices for initial calibration and sanity checking of wireless sensors. Future work might evaluate similar calibration procedures applied to other modalities like contactless heart rate or breathing rate sensing. In addition, it would be very interesting to further investigate the found activity outside to activity inside ratio in larger populations of community-dwelling older adults.

## Conclusion

We found that using calibration data from a wearable accelerometer, collected over 7–14 days, significantly improves physical activity estimates of wireless passive infrared sensor systems. This leads also to significantly stronger correlations with health indicators and outcomes, known to be associated with physical activity. Bayesian methods like Gaussian process regression, that work well with small datasets and provide an inherent predictive distribution, which can help in diagnosing when a calibration function deteriorates over time—for instance due to changes in a person's behavior. Time-spent outside should be imputed with the average activity throughout the same time period at home, multiplied by an individual outing factor. If an individual outing factor is not available, a factor of ~1.4 may be used.

We conclude that using even relatively small amounts of wearable based ground-truth data over 7–14 days, PIR based wireless sensor systems can be calibrated to give largely better estimates of older adults' daily physical activity. This increase in performance translate directly to stronger correlations with a variety of age relevant health indicators and outcomes known to be associated with physical activity.

## Data Availability Statement

The raw data supporting the conclusions of this article will be made available by the authors, without undue reservation.

## Ethics Statement

The studies involving human participants were reviewed and approved by the Kantonale Ethikkommission des Kantons Bern, Murtenstrasse 31, 3010 Bern (KEK-ID: 2016-00406). The patients/participants provided their written informed consent to participate in this study.

## Author Contributions

NS, HS, PB, PU, RM, and TN designed and planned the study. NS and HS installed and maintained the system and measured the participants. NS and AB analyzed the data. NS, AB, and HS wrote the manuscript. All authors reviewed and approved the final manuscript.

## Conflict of Interest

PB was employed by Domo-Safety SA, which is the manufacturer of the displayed sensor system. The remaining authors declare that the research was conducted in the absence of any commercial or financial relationships that could be construed as a potential conflict of interest.

## Figures and Tables

**Figure A1:**
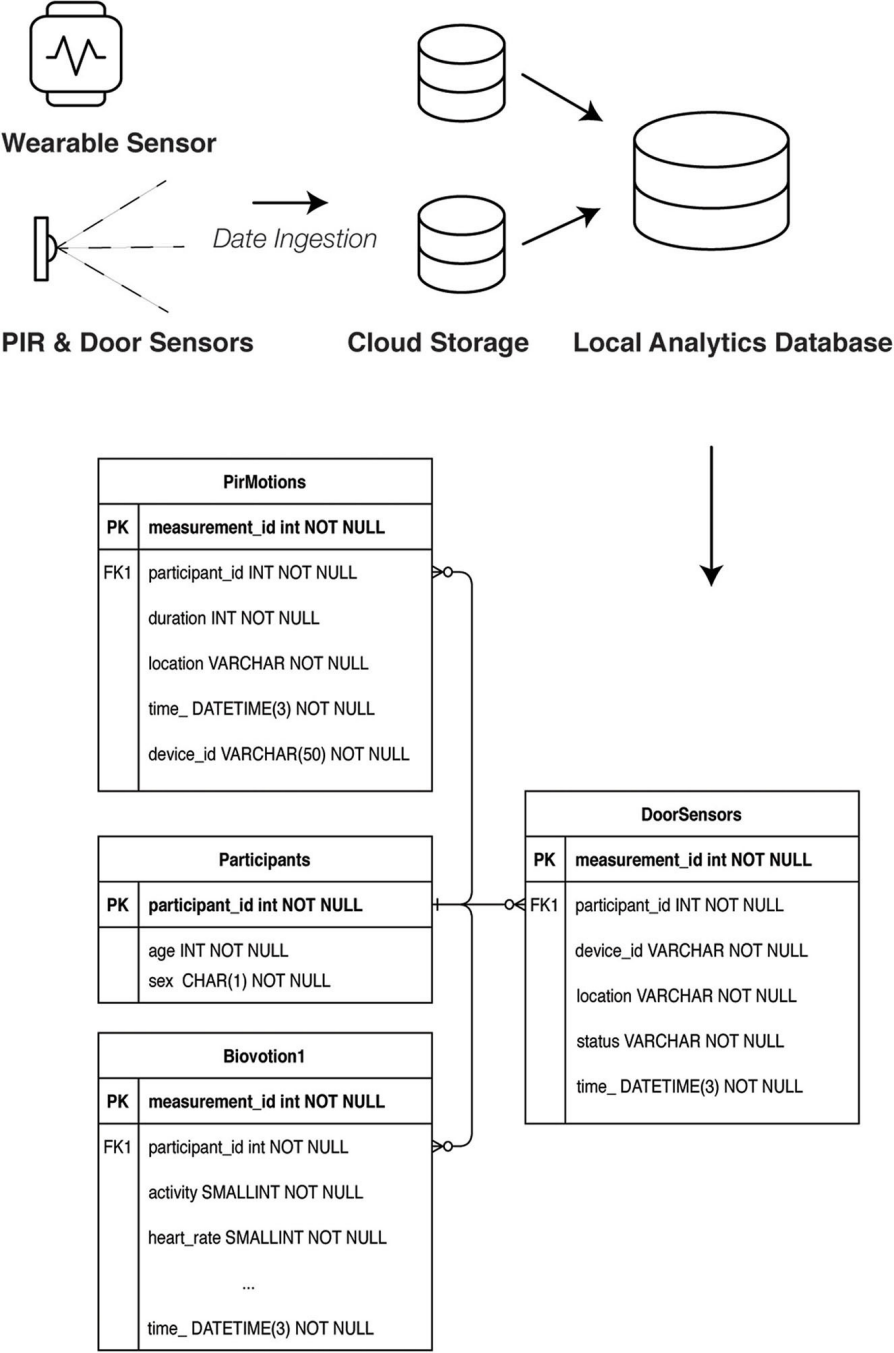
Schematic of sensor data acquisition and final data structure.

## Appendix

While most fields are self-explanatory, we describe some details regarding fields used in the calibration procedure. The “duration” attribute of the PirMotions table refers to how many seconds a given sensor was reporting motion. The location attribute of the same table describes the room the sensor was in and the time_ the exact time of the firing (in UTC). The activity field of the Biovotion1 table represents the normalized activity values stemming from the device's accelerometer and the time_ describes the exact measurement time (in UTC). The DoorSensors table's location field refers to the location the sensor was placed—in this work only entrance sensors were relevant. The status attribute describes whether the door was opened or closed and the time_ attribute denotes the exact time of this event (in UTC).
